# Application of intraoral scanner to identify monozygotic twins

**DOI:** 10.1186/s12903-020-01261-w

**Published:** 2020-10-02

**Authors:** Botond Simon, Laura Lipták, Klaudia Lipták, Ádám Domonkos Tárnoki, Dávid László Tárnoki, Dóra Melicher, János Vág

**Affiliations:** 1 Department of Conservative Dentistry, Semmelweis University, Szentkirályi, utca 47, Budapest, H-1088 Hungary; 2 Faculty of Dentistry, Semmelweis University, Budapest, Hungary; 3 Medical Imaging Center, Semmelweis University, Budapest, Hungary; 4 Hungarian Twin Registry, Budapest, Hungary; 5 Department of Obstetrics and Gynecology, Semmelweis University, Budapest, Hungary; 6 MTA-SE Immunproteogenomics Extracellular Vesicle Research Group, Budapest, Hungary

**Keywords:** Palatal rugae, Intraoral scanner, Monozygotic twin, Forensic odontology, Human identification, Palate

## Abstract

**Background:**

DNA base identification is a proper and high specificity method. However, identification could be challenged in a situation where there is no database or the DNA sequence is almost identical, as in the case of monozygotic (MZ) twins. The aim of this study was to introduce a novel forensic method for distinguishing between almost identical MZ twins by means of an intraoral scanner using the 3D digital pattern of the human palate.

**Methods:**

The palatal area of 64 MZ twins and 33 same-sex dizygotic (DZ) twins (DZSS) and seven opposite-sex dizygotic twins (DZOS) were scanned three times with an intraoral scanner. From the scanned data, an STL file was created and exported into the GOM Inspect® inspection software. All scans within a twin pair were superimposed on each other. The average deviation between scans of the same subject (intra-subject deviation, ISD) and between scans of the two siblings within a twin pair (intra-twin deviation, ITD) was measured. One-sided tolerance interval covering 99% of the population with 99% confidence was calculated for the ISD (upper limit) and the ITD (lower limit).

**Results:**

The mean ISD of the palatal scan was 35.3 μm ± 0.78 μm. The calculated upper tolerance limit was 95 μm. The mean ITD of MZ twins (406 μm ± 15 μm) was significantly (*p* < 0.001) higher than the ISD, and it was significantly lower than the ITD of DZSS twins (594 μm ± 53 μm, *p* < 0.01) and the ITD of DZOS twins (853 μm ± 202 μm, *p* < 0.05).

**Conclusion:**

The reproducibility of palatal intraoral scans proved to be excellent. The morphology of the palate shows differences between members of MZ twins despite their almost identical DNA, indicating that this method could be useful in forensic odontology.

## Background

DNA base identification is a proper, high specificity method in most situations [1]. However, identification could be challenged in a situation where there is no database, or the DNA sequence is almost identical, as in the case of monozygotic twins (MZ). There are approximately 91.7 million twins in the world, and 28 million of them are monozygotic [2]. Identification of an MZ twin still poses obstacles in forensic science [3]. In addition to DNA-based identification, phenotypic differences could be recorded, such as by facial recognition. However, MZ twins resemble each other very much in most cases; therefore, it might be challenging to tell them apart [4].

Further identification methods include recording external features of the body by two- or three-dimensional scanning, which can be complicated because the soft tissue covering the body dynamically changes due to its plasticity. An automatic high accuracy recognition method requires a stable object over time and during measurement. Facial characteristics may also be changed by an accident, plastic surgery, aging, disease, change in weight, etc. [5, 6]. Fingerprints are not identical but very similar between twins [7]. Fingerprints can be easily ruined by accidents, such as fire or water, or on purpose [8]. Thus, even small damage could prevent identification. Furthermore, a fingerprint database is not always available. Hard tissues are relatively stable at a certain age and show no plasticity [9]. Nevertheless, bone imaging requires X-ray radiation, which is not always possible due to ethical reasons [10].

Palatal rugae patterns have been studied for personal identification in the field of forensic odontology, and it has been suggested that a considerable difference exists between subjects [11]. Orthodontic treatment and extraction affect some parts, but not all, and the shape of the rugae remained constant during orthodontic treatment [12]. The anterior part of the palate is well protected by the teeth and the maxillary bone, the buccal pad of fat, the lips, and the neurocranium [13]. It is little affected after a severe burn for at least 7 weeks, both in cadavers and in patients [14]. The palatal rugae and their role in forensic odontology should be revisited as a reliable method of human identification due to the development of digital dentistry [15]. The three-dimensionally digitalized palate makes it possible to accomplish geometrical measurements with high accuracy [16, 17] and to develop an automatic pattern recognition method by artificial intelligence. The superimposition of palatal scans and the calculation of surface deviation could eliminate the former approach when the identification was made by the visual classification of palatal rugae [18–20]. Previously, intraoral scanners (IOSs) were primarily used for single crown restorations [21]. The scans for a single crown rarely included the palatal soft tissue. However, improved speed and accuracy make IOSs suitable for a long span or even full-arch prosthetic work [22, 23]. IOS is gaining popularity for making orthodontic appliances [24], measuring distances on the digital model [17], and performing orthodontic diagnosis [25]. The reliability of IOS for making full dentures has been recently suggested [26]. These applications frequently or inevitably include the palatal area; therefore, data are continually generated. The scans can be exported from dedicated software as open STL files. A complete case takes up around 100 MB space on hard drives; therefore, long-term storage could not be a problem compared to the plaster model. Furthermore, dentists frequently use online databases to share the data for orthodontic purposes, implant registration, and smile design. This will result in a rapidly growing digital database, which can be easily used for forensic purposes. The precision of IOSs for palatal digital impressions has been investigated recently, and it is found to be between 69 and 117 μm, depending on the applied IOS [27].

We proposed a method using an IOS to record the full palate for human identification. The uniqueness of forensic features is often criticized because studies are not conducted in a proper manner, or a small subject population is used [28]. To overcome this problem, we assumed that people who resemble each other as much as possible (we think of identical twins) would be the best subjects because if they can be separated by the new method, palatal morphology can be accepted as a unique trait (characteristic of an individual, clearly distinguishing an individual). As far as we are concerned, MZ twin pairs have the most phenotypic similarity. The question is whether the morphology of the human palate could differentiate between siblings of MZ pairs and whether IOSs are reliable enough to detect these small differences.

The primary aim was to determine the reproducibility of palatal scans. The secondary aim was to assess the deviation of palatal scans between siblings within MZ twin pairs. The tertiary aim was to estimate the probability of distinguishing between MZ siblings by the calculation of tolerance limits.

## Methods

### Study subjects

Two hundred and one asymptomatic twin participants, including nine same-sex triplets (147 females and 54 males), were selected from the Hungarian Twin Registry (HTR) database [29]. Each participant received written information about the subsequent measurements, enabling them to give written informed consent. The study was carried out under the Declaration of Helsinki. Ethical approval was granted on July 26, 2018 by the National Health Registration and Training Center (approval number: 36699–2/2018/EKU). Zygosity was determined by a standardized questionnaire which has nearly 99% accuracy [30, 31]. DZ pairs were also included for comparison. DZ twins share approximately 50% of their genetic material, while MZ twins are almost 100% identical regarding their genetics. One MZ pair was excluded from the analysis because we failed to make a proper palatal scan. This twin pair has Marfan syndrome with a highly arched palate [32]. The zygosity distribution of the pairs was the following: 64 MZ, 33 same-sex DZ (DZSS), and seven opposite-sex DZ (DZOS). Each triplet had one MZ pair and a DZ sibling. Therefore, one MZ comparison and two DZ comparisons were made within each triplet. The MZ triplet pairs were included in the MZ groups, whereas the DZ ones in the DZ groups. The twins were aged between 17 and 74 years (the mean age was 32 years with a standard deviation of 14.5 years). The first-born sibling was denoted by letter A and the second-born sibling by letter B. In the case of triplets, the third-born sibling was denoted by letter C.

### Data acquisition

**Fig. 1:**
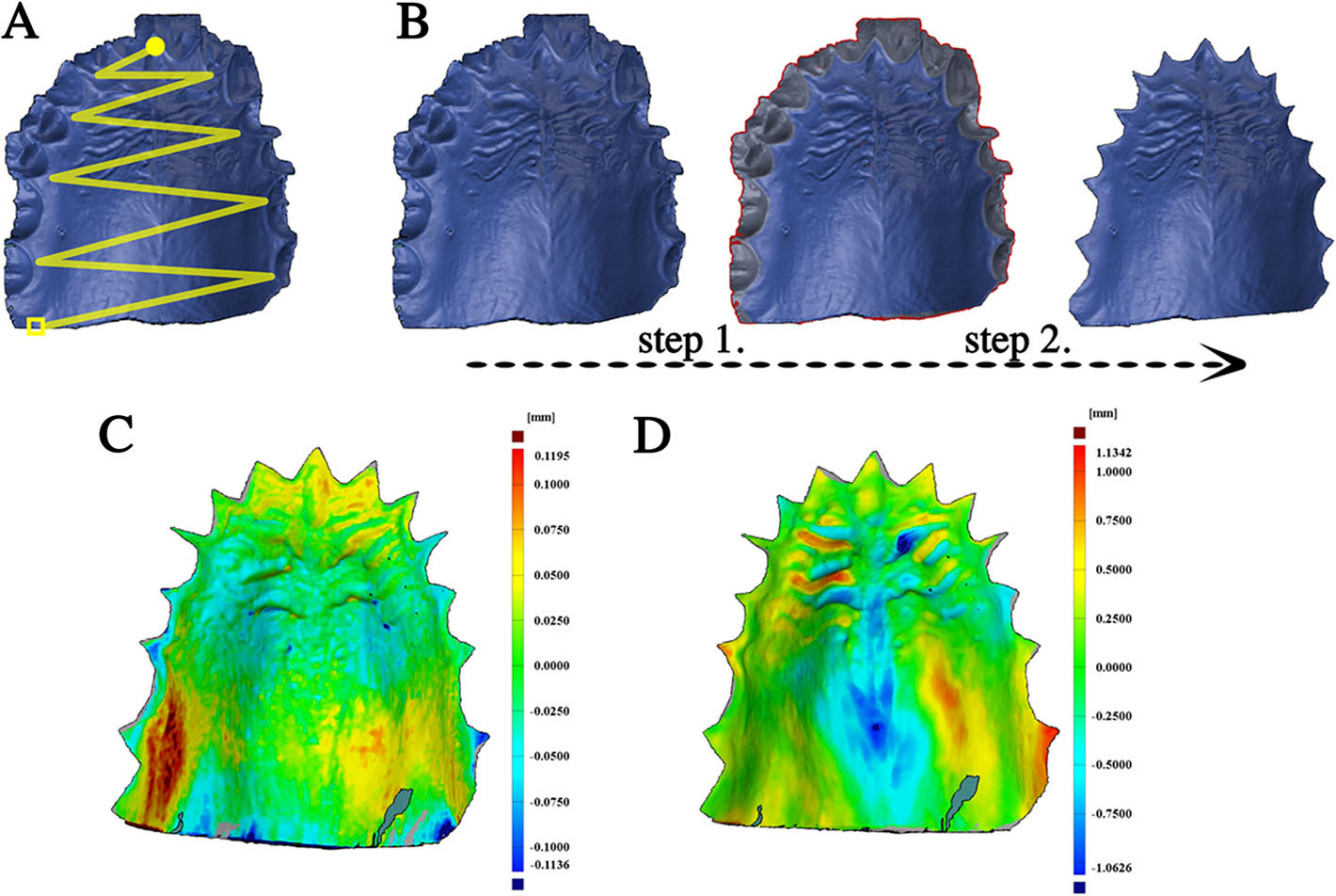
The standard scanning pattern of data acquisition was demonstrated on the upper left side (**a**). The scan was started by a zig-zag movement from the incisive papilla (yellow circle) and finished at the border of the hard and soft palate (yellow box). The preparation of the STL file was demonstrated on the upper right side (**b**). Teeth were selected (step 1) and removed (step 2) from the scan, and only the palatal area was kept for alignment and surface comparison. The result of the surface comparison with the ISD method of an MZ subject was demonstrated on the lower left side (**c**), and with ITD method of an MZ twin pair was demonstrated on the lower right side (**d**). There is one order of magnitude difference between the two scales

The palatal area of each subject was scanned with an Emerald® intraoral scanner (Planmeca Oy, Helsinki, Finland, software version Romexis 5.2.1) by a zig-zag scanning pattern (Fig. 1/a), starting from the incisive papilla and finishing at the border of the hard and soft palate. This scan was repeated three times (R1, R2, R3). The same dentist who was experienced in this specific system made all scans.

### Alignment methods and surface comparison

Each scan was exported as an STL (standard triangulation language) file into the GOM Inspect® software (GOM GmbH, Braunschweig, Germany) for data evaluation and surface comparison. Before the alignments were made, the teeth were cut off from the replicates (Fig. 1/b). Two types of alignments were made using the iterative closest point algorithm [33]. First, each scan of the same subject was aligned to each other, and mean surface deviations for the three alignments were calculated (intra-subject deviation, ISD) as shown in Fig. 1/c. Second, the mean deviation between replicates of different siblings within a twin pair was calculated (intra-twin deviation, ITD) as shown in Fig. 1/D, making nine measurements for each pair. The deviation was calculated after surface comparison. The integrated absolute distance and the area of the valid distance between the two surfaces were calculated and transferred to an Excel file, and the mean deviation was calculated as the ratio of these two parameters in order to get the absolute mean surface deviation.

### Statistical analysis

Data in the text and figures are indicated by the mean ± standard error of the mean (SE). Deviation values showed right-skewed distribution and heteroscedasticity. The variance for ISD and the comparison between ISD and ITD and between mono- and dizygotic ITD were determined by the generalized linear mixed model with gamma distribution and log-link function in SPSS 25 (IBM SPSS Statistics for Windows, Version 24.0). Variances estimated from the model were used to determine a one-sided tolerance interval (upper limit for the ISD and lower limit for the ITD) covering 99% of the population with 95% confidence (alpha level) at least without overlap of the two populations [34].

**Fig. 2:**
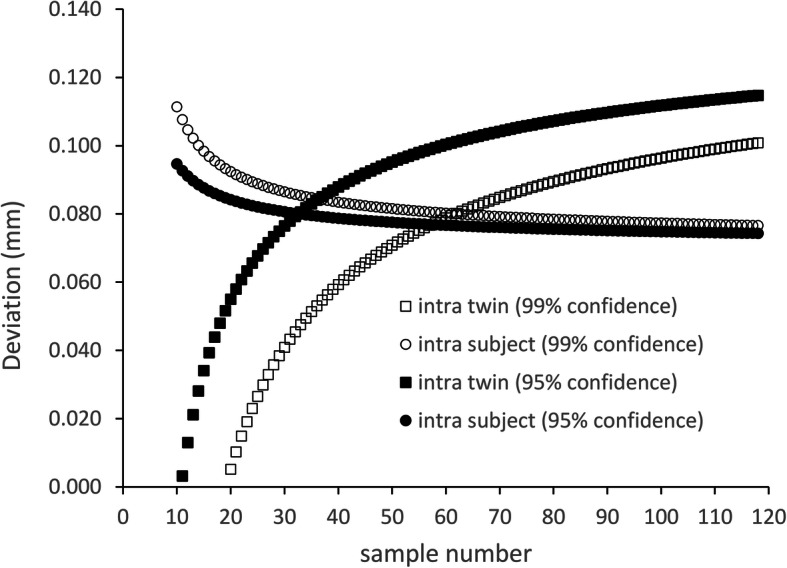
Estimation of sample size for discriminating the intra-subject (box) and the intra-twin (circle) deviation values at the 99% percentile of the population with 95% confidence (filled markers) or with 99% confidence (empty markers)

For sample size estimation, the result of a pilot experiment was used involving 22 MZ twin pairs. The mean of ISD was 34 with a standard deviation of 15 μm, and the mean of ITD was 361, with a standard deviation of 93 μm. Tolerance intervals were calculated for a range of sample numbers from 10 to 120, and the result was depicted in Fig. 2. The sample number of 33 was deemed to be eligible to separate the two groups with 99% population coverage with 95% confidence (alpha), and 66 MZ pairs were deemed to be enough to separate them with 99% confidence.

## Results

**Fig. 3:**
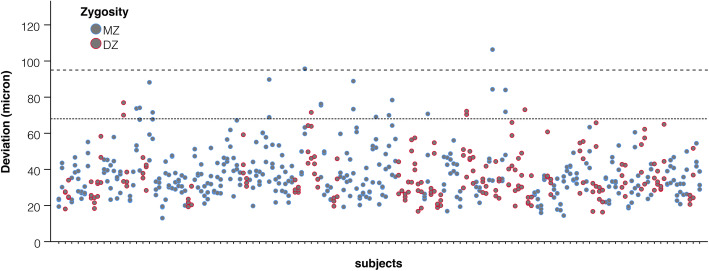
Deviations between scans within a subject (reproducibility) of MZ (blue dots) and DZ (red dots) twins. The lower dashed line indicates an upper 99% tolerance interval with 99% confidence (68 μm). The upper dashed line indicates the upper 99.999% tolerance interval with 99% confidence (95 μm)

With 199 subjects (including all types of twins), the mean ISD of the palatal scan was 35.3 μm ± 0.78 μm. No differences in ISD were observed between MZ and DZ twins (36.2 ± 0.9 vs. 34.5 ± 1.2, *p* = 0.271). The calculated upper tolerance interval was 67 μm with 99% coverage and with 95 confidence, it was 68 μm with 99% coverage and with 99 confidence, and it was 95 μm with 99.999% coverage and with 99 confidence (Fig. 3).

**Fig. 4:**
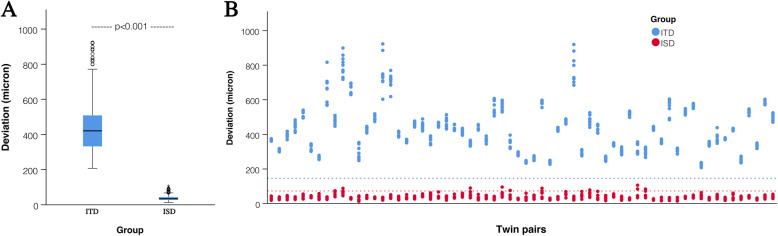
The difference in the mean (horizontal line), standard deviation (box), and the 95% confidence intervals (whiskers) (**a**) and the dispersion of individual values (**b**) between the intra-subject (ISD) and intra-twin group (ITD). The lower dashed line indicates the upper 99% tolerance interval with 99% confidence (73 μm) of the intra-subject deviation values. The upper dashed line indicates the lower 99% tolerance interval with 99% confidence (138 μm) of the intra-twin deviation values

The superimposition of two scans of the same subject (Fig. 1/c) always resulted in a smaller deviation value than the deviation between two scans within siblings of the same MZ pair (Fig. 1/d). The mean ITD of the 64 MZ twins was significantly higher than the ISD values (411 ± 15.2 μm vs. 37 ± 1.1 μm, *p* < 0.001) (Fig. 4). The calculated lower 99% tolerance limit (with 99% confidence) of the ITD of MZ twins was 147 μm, and the upper 99% tolerance interval of ISD was 73 μm with 99% confidence (Fig. 4).

**Fig. 5:**
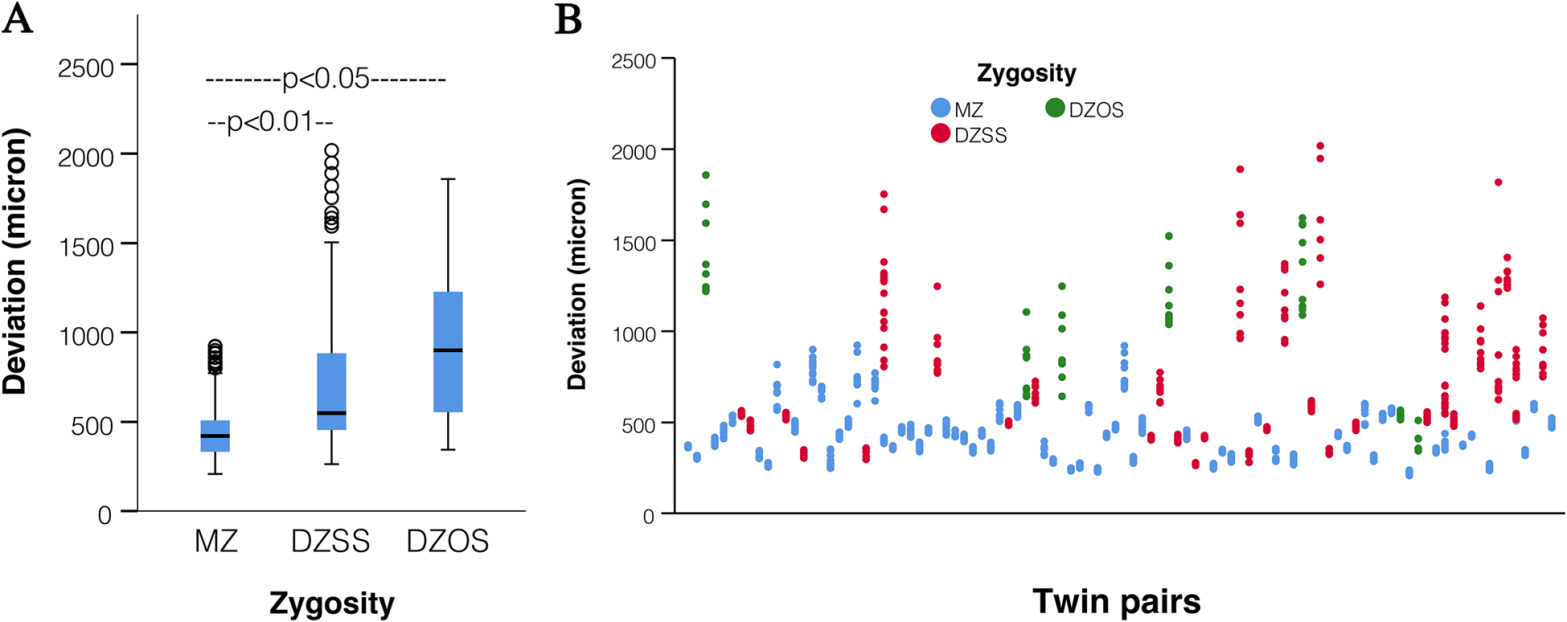
The difference in intra-twin deviations (ITD) between monozygotic (MZ) and dizygotic (DZSS and DZOS) twins. The mean (horizontal line), standard deviation (box), and 95% confidence intervals (whiskers) (**a**) and dispersion of individual values (**b**) are shown

The mean ITD of MZ twins (406 ± 15 μm) was significantly lower than that of DZSS twins (594 μm ± 53 μm, *p* < 0.01) and that of DZOS twins (853 μm ± 202 μm, *p* < 0.05). No significant difference was observed between DZSS and DZOS twins (Fig. 5).

In MZ twins, a weak but significant correlation (*r* = 0.3, *p* < 0.05) was found between the ITD and the subject’s age. From the regression equation, the mean ITD was 384 μm at age 17, and it increased by 3 μm every year. No correlation was found in the case of the ITD in DZ twins or in any of the ISD cases.

## Discussion

This study aimed to assess the reproducibility (precision) of palatal intraoral scans in order to distinguish between almost identical twins based on palatal morphology. The reproducibility of palatal intraoral scans was assessed by calculating surface deviation between scans of the same individuals. An ISD of 35.3 μm is better than what was found in previous studies [27, 35], providing a range between 55 and 117 μm. We aimed to calculate the upper tolerance limit of ISD with 99% coverage of the population and with 95% confidence. The lower boundary means that 99 measurements out of 100 are expected to be below this level with 95% certainty. Due to the relatively large sample size and the high precision of the palatal scans, we were able to increase both population coverage and confidence to 99.999 and 99%, respectively. It means that out of 100,000 ISD measurements, 99,999 will result in less than 95 μm. This assessment was done with three replicates. In forensic science or forensic identification, at least two scans are necessary for control purposes. After aligning the two scans, it is recommended to make a new scan if the measured deviation value is higher than 95 μm. A palatal scan can be completed in 18 to 22 s, and the alignment takes another minute.

Among the investigated 64 MZ pairs, the smallest deviation between two scans acquired from two different siblings of the same MZ pair was 208 μm. In contrast, the highest intra-subject deviation was 106 μm. There was no overlap between the two populations. The calculation of tolerance intervals allows us to estimate values for the whole population. Based on our findings, the ITD values can be separated from the ISD values at a 99% tolerance limit with 99% confidence. The lower limit of ITD (138 μm) and the upper limit of ISD (73 μm) do not overlap. This result suggests that the morphology of the palate between members of MZ twin pairs is different despite their almost identical DNA sequence. The intraoral scans made with the Emerald intraoral scanner on palatal soft tissues are reliable to differentiate within MZ twin pairs. According to our results, if we compare two unidentified intraoral scans and the measured deviation value is higher than 138 μm, we can be 99% certain that these two scans are not from the same subject, even if that subject has an MZ twin brother or sister.

Using a palatal scan for identification in forensic odontology has distinct advantages. The palatal area remains more or less intact through life compared to the teeth, which continually change due to dental treatment. It is less vulnerable to external impact than other external surface structures, such as fingerprints [13, 14]. The 3D evaluation of landmarks of the palatal rugae showed no significant changes over 2 years [36]. Notably, the horizontal dimension of the maxillary arch was not different from 13 to 45 years of age [37]. Presumably, palatal morphology may also remain unchanged. However, based on our results, the difference between siblings within an MZ pair slightly increases with age. This observation can be explained with different epigenetic changes evolving in MZ twins as they grow older [38]. Over 50 years, the difference between two MZ twins increases by about 150 μm on average, and it increasingly approaches the mean deviation between two DZ twins. The increased deviation may be due to the involution of the gingiva after tooth loss. The higher deviation favors the accuracy of identification. The superimposition of the palate could be a reliable method for identifying a person, which was suggested in a previous study [36] as well.

However, the method may have some limitations. Some orthodontic treatments, such as the rapid maxillary expansion of the palate, could distort its morphology [39]. Sparse data suggest that distances between the palatal rugae may change after graft harvesting [40]. No information is yet available about the postmortem laceration of the palate after drowning, the regeneration of its pattern after graft harvesting for periodontal surgery, and a possible change due to tissue atrophy during aging and tooth loss. Therefore, further studies should investigate these underlying questions.

Further limitations of our novel method are the lack of an accuracy study of palatal scans using different IOSs. In two clinical studies, the reference model was made with a conventional (polyvinyl siloxane) impression. The trueness of the palatal area scanned with Trios 3 was 80.5 μm [16] when it was superimposed with the reference model. In an earlier study [35] with a similar reference impression and IOS, the trueness of the palate was 130.5 μm. This suggests an improvement in the accuracy of the scanners over time. However, the reference model made by a conventional impression could distort the trueness value. In a cadaver, the error could be eliminated if the dislocated maxilla can be scanned directly by a highly accurate industrial scanner to get a reference model [27]. It has been the only study so far where the accuracy of the palate scan was evaluated using the Emerald scanner. The trueness and precision were 150.6 μm and 87.1 μm, respectively. However, only the teeth were aligned before the deviation in the palatal region was determined. It is a rational assumption that if a region other than the one measured is aligned, then the best-fit alignment would not be optimized for that region. Alignment at the palatal region instead of the teeth may explain the higher precision (35 μm) in our study.

The trueness of 150 μm measured in the cadaver study [27] is much lower than the mean ITD value (411 μm) of MZ twins in our study and is very close to the lower 99% tolerance limit of the ITD of MZ twins (147 μm). If the patient was previously scanned by a scanner other than that used during the identification process, additional errors might be introduced. Notably, errors are always additive at the variance (square of the standard deviation) level; thus, the result is somewhat lower than the sum of the trueness values. Furthermore, a previous study [27] demonstrated that contrary to the conventional impression, all investigated IOSs had positive deviation values, suggesting that the deviation from the true value was in the same direction, which may further decrease the discrepancy between scanners. Automated human identification became feasible when digital impressions will become widespread, and by then, there is a good chance that the accuracy of IOSs will also improve significantly. Furthermore, we assumed that MZ twins were the most resembling subjects; thus, much more deviation was expected between non-twin people.

## Conclusion

In conclusion, the superimposition of intraoral scans of the palate could be a quick, easy, and highly reliable way of human identification. Monozygotic twin siblings can be distinguished from each other with high confidence, and it might imply uniqueness for the whole human population.

## Supplementary information

**Additional file 1.**

## Data Availability

The datasets used and/or analyzed during the current study are available from the corresponding author on reasonable request.

## Abbreviations

MZ: Monozygotic
DZ: Dizygotic
DZSS: Same-sex dizygotic twins
DZOS: Opposite-sex dizygotic twins sex
ISD: Intra-subject deviation
ITD: Intra-twin deviation
IOS: Intraoral scanner
HTR: Hungarian twin registry
STL: Standard triangulation language
SE: Standard error of the mean
