# Supplementary material for: Application of intraoral scanner to identify monozygotic twins
Source: BMC Oral Health. 2020 Oct 2;20:268. doi: 10.1186/s12903-020-01261-w (PMC7532102; doi:10.1186/s12903-020-01261-w)

**Application of intraoral scanner to identify monozygotic twins**

Botond Simon 1, Laura Lipták 2, Klaudia Lipták 2, Ádám D. Tárnoki 3,6, Dávid L. Tárnoki 3,6, Dóra Melicher 4,5,6, János Vág 1

**Affiliation**

1 Department of Conservative Dentistry, Semmelweis University, Budapest, Hungary

2 Faculty of Dentistry, Semmelweis University, Budapest, Hungary

3 Medical Imaging Centre, Semmelweis University, Budapest, Hungary

4 Department of Obstetrics and Gynaecology, Semmelweis University, Budapest, Hungary

5 MTA-SE Immunproteogenomics Extracellular Vesicle Research Group

6 Hungarian Twin Registry

corresponding author: Dr. Botond Simon

mailing address: Department of Conservative Dentistry, Semmelweis University, Szentkirályi

utca 47., H-1088, Budapest, Hungary

e-mail: [dr.simon.botond@gmail.com](mailto:dr.simon.botond@gmail.com)

numbers of pages: 3

Appendix Figure 1.


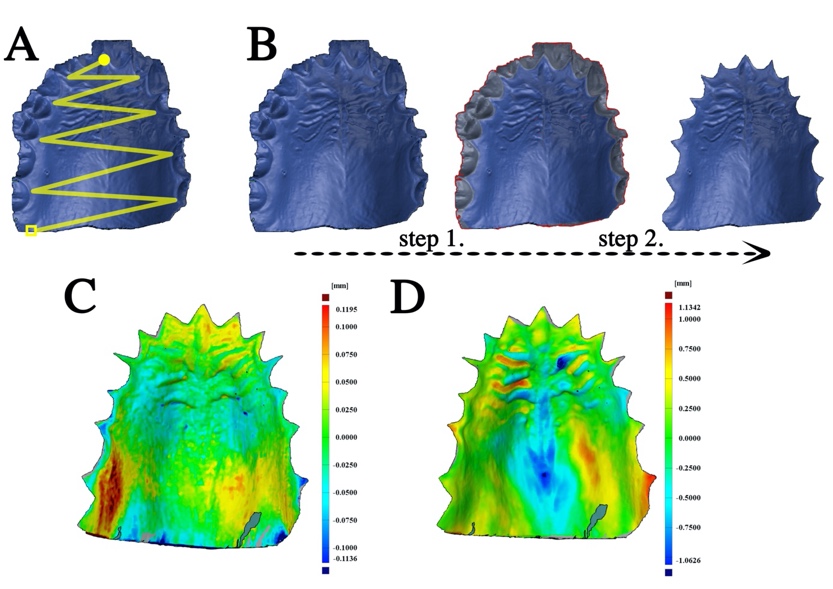


Appendix Figure 2.


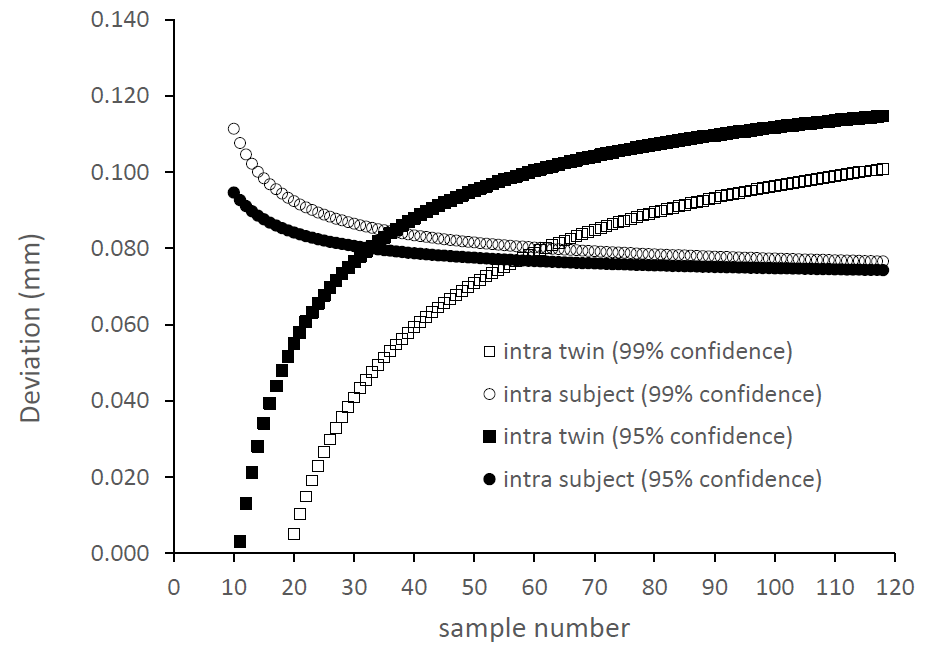


Appendix Figure 3.


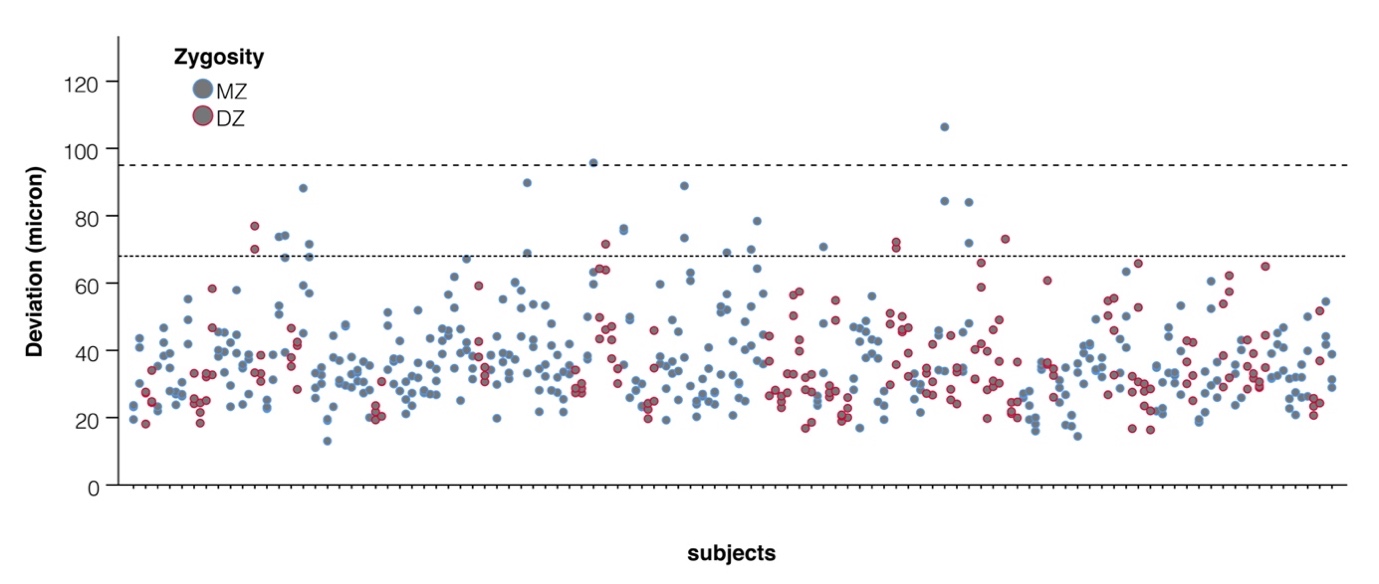


Appendix Figure 4.


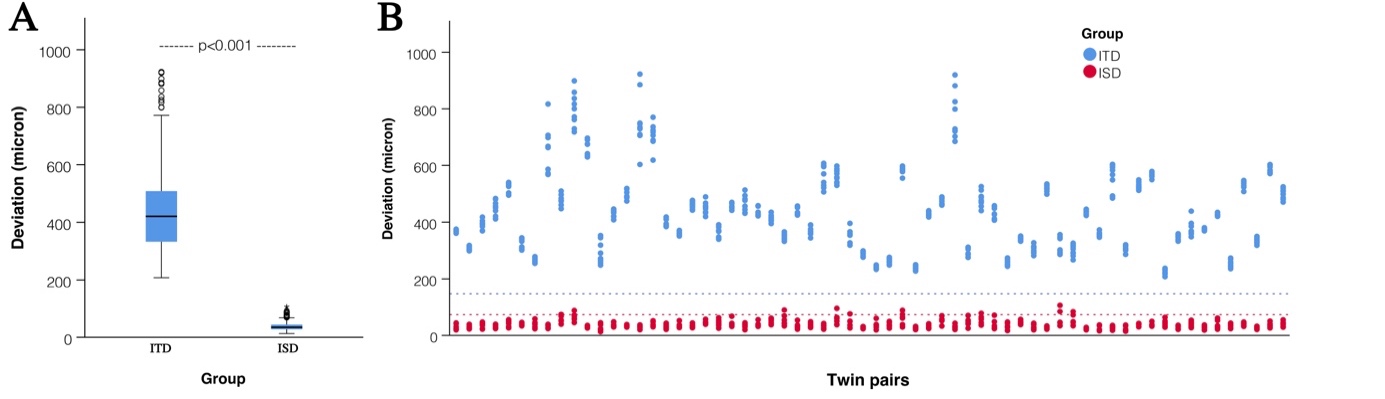


Appendix Figure 5.


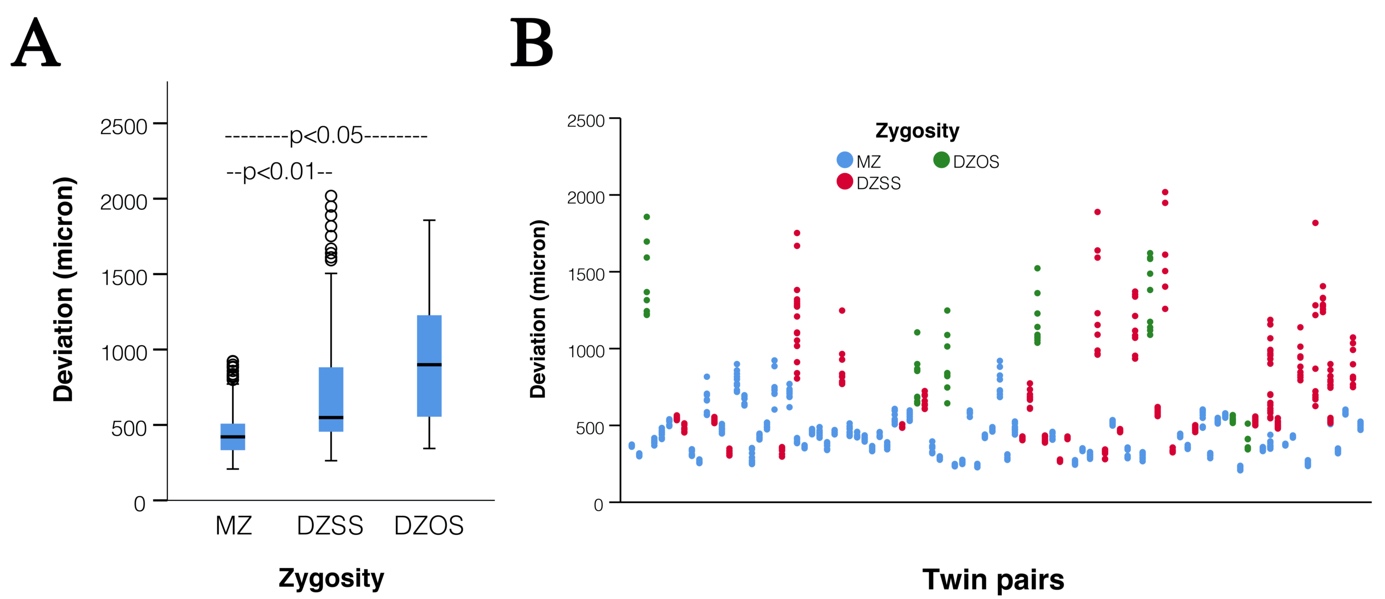

Supplement: Supplementary file 1 — Additional file 1. [file 12903_2020_1261_MOESM1_ESM.docx]
